# Pistachio Hull Extract as a Practical Strategy to Extend the Shelf Life of Raw Minced Beef: Chemometrics in Quality Evaluation

**DOI:** 10.1155/2021/2429766

**Published:** 2021-08-16

**Authors:** Khaoula Elhadef, Karim Ennouri, Mariam Fourati, Hajer Ben Hlima, Sarra Akermi, Lotfi Mellouli, Slim Smaoui

**Affiliations:** ^1^Laboratory of Microbial, Enzymatic Biotechnology and Biomolecules (LBMEB), Center of Biotechnology of Sfax, University of Sfax-Tunisia, Road of Sidi Mansour km 6, P. O. Box 1177, 3018 Sfax, Tunisia; ^2^Laboratory of Amelioration and Protection of Olive Genetic Resources, Olive Tree Institute, Sfax University, Sfax, Tunisia; ^3^Laboratory of Enzyamatic Engineering and Microbiology, Algae Biotechnology Unit, Biological Engineering Department, National School of Engineers of Sfax, Sfax 3038, University of Sfax, Tunisia

## Abstract

The agricultural processing industry produces a notable quantity of by-products rich in bioactive compounds, which can be exploited for agri-food applications. From pistachio industrial processing, pistachio's hull is one of the major by-products. This work aimed to evaluate the potential of pistachio hull, as a potential source of natural antioxidant, to preserve the meat quality. Here, we investigated the impact of aqueous pistachio hull extract (PHE) at 0.156% (PHE1), 0.312% (PHE2), and 0.625% (PHE3) on the quality of raw minced beef meat stored for 14 days at 4°C. At the end of storage, mesophilic total viable plate, psychotropic and Enterobacteriaceae counts, showed significantly lower (*P* < 0.05) microbial count in PHE samples. PHE3 revealed a powerful inhibitory effect on lipid/protein oxidation, and sensory characteristics were positively (*P* < 0.05) affected. Principal component analysis and heat map indicated complex and close synchronized relations among lipid/protein oxidation processes, microbial loads, and sensory attributes. Obtained results using univariate and multivariate statistical analysis underlined the importance of using different mathematical approaches, which are complementary to each other and could provide considerable information about the minced beef meat treated by PHE. Therefore, compared to synthetic antioxidants, PHE could be a clean-label alternative that can protect and enhance the quality of meat products.

## 1. Introduction

Extending the shelf life of meat and meat products through the control of chemical processes and microbial contamination, both within and upon product surfaces, is important to guarantee that the safety, quality, and nutritional status of products are preserved throughout the distribution chain for as long as possible, effectively attaining consumers for consumption [1–3]. Nowadays, the use of antioxidants from plant matrices and their by-products emphasizes the necessity of antioxidant solutions for the meat industry since consumers perceive them as safe and are Generally Recognized as Safe (GRAS) [4, 5]. This strategy is becoming an attractive strategy in order to enhance quality- and health-related characteristics of meat products. Some plants such as *Hibiscus sabdariffa* L. [6], *Punica granatum* [7], *Ephedra alata* [8]*, Vachellia nilotica* [9], and *Ilex meserveae* [10] extracts are incorporated in different beef meat products as “natural antioxidants.” These active compounds can maintain characteristics of beef meat by retarding chemical oxidation and minimizing microbial spoilage caused by aerobic microorganisms and therefore prolonging the shelf life.

In the world, *Pistacia vera* L. is the most industrialized variety; nevertheless, it produces important quantities of by-products, which are considered as waste and can lead to environmental problems [11]. Conversely, these by-products, especially the hull, have diverse valuable phytochemical groups. Based on the chemical composition, many studies are conducted on pistachio hull extracts displaying that it contains different types of antioxidants, including anthocyanins, flavan-3-ols, proanthocyanidins, flavonols, isoflavones, flavanones, stilbenes, and phenolic acids [11, 12]. In addition, some functional properties of pistachio hull extracts have been previously studied in terms of antioxidant [13–15] and antimicrobial activity [16]. Pistachio hull extracts could be considered as a suitable additive in food industries due to the presence of valuable compounds that established multiple functional effects. Recently, Abolhasani et al. [14] and Fattahifar et al. [17] revealed that pistachio hull can be useful in prolonging browning reactions in some foodstuffs due to its the antityrosinase activity. Furthermore, the antioxidant and antimicrobial properties of pistachio hull extracts have been studied in some food formulations such as chicken burger [18]. It was also reported that it could delay soybean oil fat oxidation [15, 19] and rheological and sensory properties of marmalade [20].

Actually, continuous research studies are in progress to elaborate efficient and healthy natural substrates for application in meat preservation and in light of the multiple issues that pistachio hull extract (PHE) could resolve it. The present study investigated the benefit that PHE might provide in terms of the chemical, microbiological, and sensory attributes of minced beef meat. The study aimed to understand the links between quality parameters and analyses by PCA and heat map in order to provide more information about sample distribution at different storage time periods.

## 2. Materials and Methods

### 2.1. Plant Material and Extraction

Pistachio (*Pistacia vera* L) hulls were harvested and sampled in August 2020 from farms located in Sfax (N: 34.4426°, E: 10.4537°). The hulls were ground to a fine powder using an electric grinder (Moulinex, France). Then, the powder was extracted by mixing with distilled water (ratio of 1 : 8) and stirring overnight at room temperature using a magnetic stirrer. Later, all the samples were centrifuged (Sorvall Biofuge Stratos, ThermoScientific, Hanau, Germany) at 12,000x for 20 min. The aqueous supernatant was freeze-dried (Martin Christ, Alpha 1–2 LD plus Germany) and stored at −20°C for more application and analysis.

PHE was used as biopreservative in minced beef for its richness in phenolics.

Antioxidant and antimicrobial properties of PHE were previously demonstrated by Elhadef et al. [13]. Taking into account its originality, total phenolic content (TPC), total flavonoid content (TFC), total tannin content (TTC), and total anthocyanin content (TAC) are 182.11 mg GAE/g, 15.54 mg QE/g, 68.24 mg CE/100 g, and 40.98 *µ*g cyanidin-3-O- glucoside/g, respectively. Regarding antioxidant activity, evaluated by ABTS and DPPH scavenging assays, concentrations providing 50% of radical scavenging activity (EC50) of PHE are 0.19 and 0.09 mg/mL. For antibacterial activity, assessed by the agar diffusion method and evaluated by measuring the diameters of circular inhibition zones around the wells, PHE displayed the diameters of inhibition zones 14.5, 17.25, 16.25, 14.25, 14.25, and 15.75 mm, respectively, against *S. aureus*, *L. monocytogenes*, *B. cereus*, *S. enterica*, *E. coli,* and *P. aeruginosa*, respectively [13].

### 2.2. Preparation of Minced Beef Meat Samples

We bought fresh beef meat from a regional slaughterhouse located in Sfax. The beef meat was grounded using a sterile meat grinder. Then, we divided the raw minced beef meat in five lots: lot 1 and lot 2 were used as controls (lot 2 was supplemented with BHT at 0.01%), PHE was added to the minced beef at three concentrations equivalent to MIC (0.156% (*v*/*w*) (PHE1)), 2 × MIC (0.312% (*w*/*v*) (PHE2)), and 4 × MIC (0.625% (*w*/*v*) (PHE3)) against *L. monocytogenes* ATCC19117 [13]. We followed the same protocol described by Elhadef et al. [8] to make a homogeneous mixture of each treatment and then we kept them under vacuum using plastic bags to contribute three replicates. Finally, all aliquots were saved for 14 days at 4°C ± 1°C, and quality characteristics were analyzed in days 0, 3, 7, 10, and 14.

The total number of analyzed samples was 225 (75 × 3). For microbiological physicochemical and sensory tests, 75 trials (5 × 3 × 5) were used, obtained as follows: five treatments (C, BHT, PHE1, PHE2, and PHE3) for three subsamples and for each ageing period (five storage periods: 0, 3, 7, 10, and 14 days).

### 2.3. Analysis of Meat Samples

#### 2.3.1. Microbiological Analysis

25 g samples were mixed in 225 mL of sterile 0.85% NaCl solution for 10 min. The aerobic plate count (APC) was enumerated on plate count agar (PCA, Oxoid, UK) incubated at 30°C for 48 h [21, 22]. The aerobic psychrotrophic count (PTC) was determined on plate count agar (PCA) incubated at 7°C for 10 days [23]. Enterobacteriaceae count was enumerated on violet red bile glucose medium (VRBG, Oxoid, UK), incubated at 37°C for 24 h [24]. Results were calculated and expressed as log_10_ CFU (colony forming units)/g of meat.

#### 2.3.2. Physicochemical Analysis

*(1) Protein Oxidation*. Protein oxidation was estimated in terms of formation rate of metmyoglobin (MetMb %) and carbonyl groups:

(1) *Metmyoglobin (MetMb %).* MetMb was evaluated following the procedure described by Krzywicki [25]. 5 g of each aliquot was mixed with 25 mL of 0.04 M K_3_PO_4_ buffer (pH 6.8). Homogenates were held in an ice bath for 1 h to process complete extraction and centrifuged at 4500 ×g for 30 min. Finally, the absorbance was calculated at 525 (A525), 572 (A572), and 700 (A700) nm. The MetMb% was quantified using the following equation:
  (1) $$\begin{array}{c} \text{MetMb}\%=\left[-2.51\left(\frac{A_{572}}{A_{525}}\right)+0.777\left(\frac{A_{565}}{A_{525}}\right)+0.8\left(\frac{A_{545}}{A_{525}}\right)+1.098\right]\times 100. \end{array}$$
(2) *Determination of Carbonyls*. Carbonyl groups were detected by their reactivity with 2,4-dinitrophenylhydrazine (DNPH) to form protein hydrazones following the method of Ariga [26]. 1 g was suspended in phosphate buffer (20 mM, pH 6.0) to 5 mg/mL. Two samples (400 *µ*L/each) were collected from each aliquot solution: one sample was combined with 800 *µ*L of 2 M HCl including DNPH at 0.2% (*w*/*v*); the other sample was combined with 800 *µ*L of 2 M HCl (blank sample). After that, aliquot was precipitated with 400 *µ*L of trichloroacetic acid (10%, *w*/*v*) and centrifuged at 5000 × *g* for 5 min. The pellet was homogenized with 1 mL of ethanol-ethyl acetate solution (1 : 1, *v*/*v*) and centrifuged under the same condition. This process was repeated twice. Then, 1.5 mL of 20 mM NaH_2_PO_4_ (pH 6.5) including 6 M guanidine hydrochloride was added. The absorbance was calculated at 370 nm. The protein carbonyl content was calculated in accordance with a molar extinction coefficient (22000 M^−1^ cm^−1^) and expressed in nmol carbonyl mg/protein.

*(2) Lipid Oxidation*. Lipid oxidation was estimated based on the primary lipid oxidation compound (peroxide value (PV) and conjugated dienes (CD)) and secondary lipid oxidation products (malondialdehyde):

(1) *Peroxide Value (PV).* PV was assessed according to the ISO 960:2 (2007) [27]. Fatty fraction was extracted with chloroform, and later, oxidation of potassium iodate to iodine form was done by active oxygen in the presence of acetic acid. The amount of iodine generated was then determined by volumetric titration with sodium thiosulphate, and values were expressed in meq of peroxide/kg of meat.
(2) *Conjugated Dienes Hydroperoxides (CDs)*. 0.5 g of each sample of beef meat was suspended in 5 mL of distilled water and mixed. A 0.5 mL sample of this suspension was mixed with 5 mL of extracting solution, hexane: isopropanol, at 3 : 1 (*v*/*v*) for 1 min and centrifuged at 2000 × g for 5 min. Absorbance of the supernatant was measured at 233 nm. CD was calculated using the molar extinction coefficient of 25200 M^−1^ cm^−1^, and the results were expressed as *μ*mol/mg [28].
(3) *Thiobarbituric Acid Reactive Substances (TBARS) Value.* Two grams of the sample, combined with 100 *μ*L of butylated hydroxyl toluene in ethanol (1 g/L) and 16 mL of TCA at 50 g/L, was mixed for 10 min and filtered. 2 mL of the filtrate (or 2 mL of TCA for blank) was added to 2 mL of thiobarbituric acid solution (20 mol/L). Absorbance was measured against the blank at 508, 532, and 600 nm. The absorbance was corrected for the baseline drift as follows:
  (2) $$\begin{array}{c} A_{532\text{ nm corrected}}=A_{532\text{ nm}}-\left[\left(A_{508\text{ nm}}-A_{600\text{ nm}}\right)\times \frac{\left(600-532\right)}{\left(600/508\right)}\right]-A_{600\text{ nm}}. \end{array}$$
TBARS values were expressed as mg of malonaldehyde equivalent per kg of sample (mg/kg) with the molar extinction coefficient of the MDA-TBA adducts at 532 nm (1.56 × 10^5^ M^−1^ cm^−1^) [29]. MDA was determined using the following equation:
  (3) $$\begin{array}{c} \text{MDA}\left(\frac{\text{mg}}{\text{kg of meat}}\right)=\left[\frac{A_{\text{corrected}}\times \text{VTCA}\times 2\times \text{MMDA}\times 0.01}{1.56\times \text{m}}\right]. \end{array}$$

#### 2.3.3. Sensory Evaluation

Twenty trained members of the panel conducted sensory evaluation. Sensory attributes including color, appearance, odor, and overall acceptability (OA) were assessed on days 0, 3, 7, 10, and 14 of storage at 4°C by using a 9-point hedonic scale. Attribute scales varied from 1 to 9 with 9 being very good, 5 being the limit of acceptability, and 1 being very bad. A score below 5 indicated the sample to be unacceptable [30].

### 2.4. Statistical Analysis

Measurements were done 0, 3, 7, 10, and 14 days, and experiments with five treatments were used in a randomized complete block design. All analytical determinations were performed in triplicate. A two-way analysis of variance (ANOVA) with two factors (treatments and storage time) was carried out using SPSS 19 statistical package (SPSS Ltd., Woking, UK). Means and standard deviations were calculated and a probability level of *P* < 0.05 was used in testing the statistical significance of all experimental data. Tukey's post hoc test was used to determine significance of mean values for multiple comparison at *P* < 0.05.

To group samples based on chemical oxidation microbial counts and sensory traits during storage, all variables were autoscaled prior to chemometrics application. By using XLSTAT software for Windows (v.2014.1.08, Addinsoft, New York, USA), principal component analysis (PCA) and heat maps were performed to distinguish between samples at 0, 3, 7, 10, and 14 days. For all samples, dendrograms were established to obtain a two-dimensional projection of the dissimilarity or similarity of the entire sample set.

## 3. Results and Discussion

### 3.1. Microbial Analysis

During storage time, we observed a significant growth of all microbial counts (*P* < 0.05), mainly in control and BHT samples (Table 1). Also, a significant decrease in APC growth rate (*P* < 0.05) was induced by PHE addition. The microbial spoilage of meat occurs when APC and PTC reach 6.7-log CFU/g [31]. Control samples touched limits of shelf life on the 7^th^ day; however, PHE3 reached on day 14. PTC registered for PHEs was noted to lower the detection limits until the 14^th^ day. On the other hand, PHE reduced successfully the Enterobacteriaceae counts in meat. After 14 days, PHE1, PHE2, and PHE3 delayed Enterobacteriaceae counts to 1.13, 1.63, and 1.66 log CFU/g, therefore extending the shelf life until 14 days. In the same way, Elhadef et al. [13] mentioned that aqueous pistachio hull extracts contained phenolic compounds that have an inhibitory effect on various food-borne pathogens. Furthermore, TPC, TFC, and TAC have been exceedingly associated with antibacterial activity. These authors, also, demonstrated that *Ephedra alata* aqueous extract, used at 0.156, 0.312, and 0.624%, has an antimicrobial potential on minced beef meat during its refrigeration and storage.

### 3.2. Oxidative Stability Evolution

#### 3.2.1. Protein Oxidation

**Figure 1:**
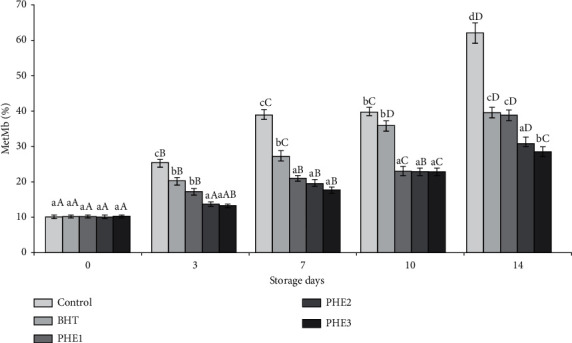
Effect of PHE on MetMb of raw minced meat beef stored at 4°C. Values with a different letter (a-b) of the same storage day are significantly different (*P* < 0.05); values with a different letter (A–D) of the same concentration are significantly different.

**Figure 2:**
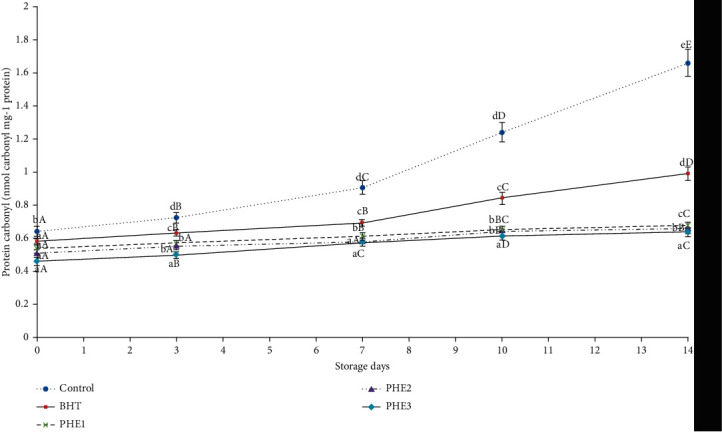
Effect of PHE on protein carbonyl (nmol carbonyl/mg of protein) of raw minced meat beef stored at 4°C. Values with a different letter (a–d) of the same storage day are significantly different (*P* < 0.05); values with a different letter (A–D) of the same concentration are significantly different.

Color is the most important factor in meat products influencing the consumer purchase decision and affecting the perception of freshness. Purchasing intent of fresh meat by consumers is based largely on Mb content of muscles and it is often implicated in its color stability. In fact, higher Mb concentrations lead to rapid oxidation and a decrease in color stability in beef muscles. The consumer rejection occurred at 40% MetMb in meat products [2]. As shown in Figure 1, antioxidants (BHT and PHE) are efficient (*P* < 0.05) in avoiding MetMb oxidation and maintaining the red color of beef meat until the 14^th^ day of storage. Direct oxidation of the side chains from Lys, Thr, Arg, and Pro can module carbonyls (ketones and aldehydes) in proteins [32]. Control and BHT samples presented significantly (*P* < 0.05) higher amounts of protein carbonyls as compared to the treated ones during sampling days (Figure 2). Similarly, the decline in carbonyl groups was previously disclosed using beef patties [33], beef meat balls [34], and minced beef meat [7, 8, 13]. Thus, protein degradation, denaturation, and loss of functionality are due to the formation of protein carbonyls from amino acid side chains caused by the impairment of myofibrillar protein conformation [35].

#### 3.2.2. Lipid Oxidation

**Figure 3:**
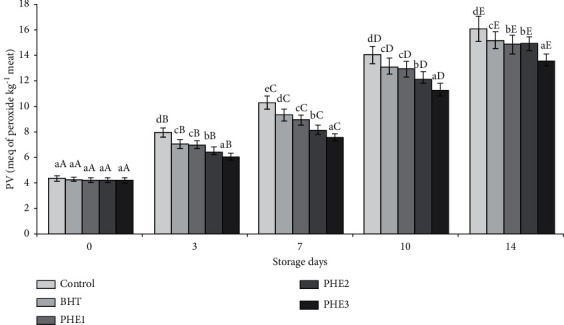
Effect of PHE on peroxide values (meq of peroxide/kg of meat) of raw minced meat beef stored at 4°C. Values with a different letter (a–d) of the same storage day are significantly different (*P* < 0.05); values with a different letter (A–E) of the same concentration are significantly different.

In order to evaluate the PHE impact on lipid oxidation, primary (PV and CD) and secondary (TBARS) product concentrations were measured. During storage, in all treatment, PV increased significantly (*P* < 0.05). Except day 0, meat samples incorporated with 0.156, 0.325, and 0.625 mg/g showed significantly lower PV than control samples (Figure 3). On the other hand, we noticed that PV did not exceed the detection limit (25 meq O_2_ kg^/^lipid), which was reported by Sallam et al. [36].

**Figure 4:**
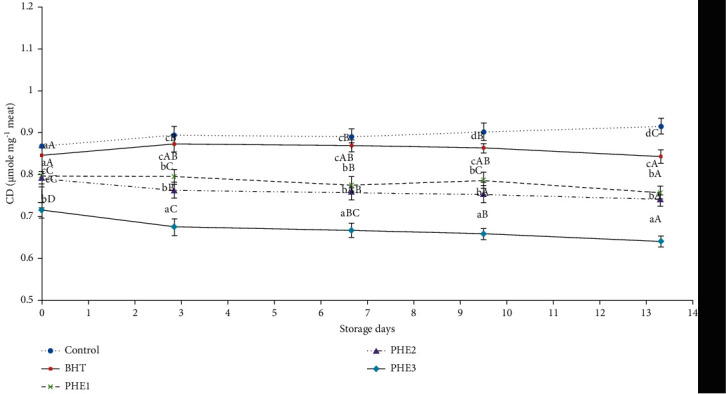
Effect of PHE on conjugated diene hydroperoxides (*μ*mole/mg meat) of raw minced meat beef stored at 4°C. Values with a different letter (a–d) of the same storage day are significantly different (*P* < 0.05); values with a different letter (A–D) of the same concentration are significantly different.

Regarding CD, formed by polyunsaturated acid oxidation, we distinguished a continuous decrease in their formation in meat treated with PHE at 0.325 and 0.625 mg/g (Figure 4). These results were in accordance with studies done by Elhadef et al. [8].

**Figure 5:**
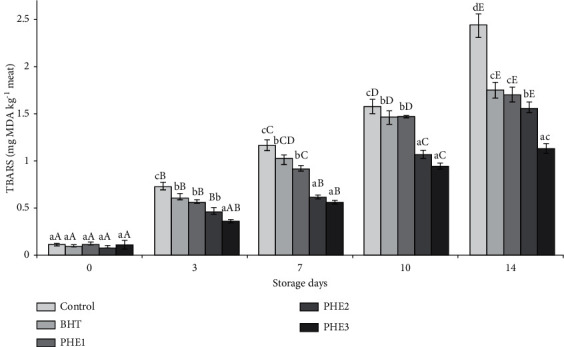
Effect of PHE on TBARS (mg/kg meat) of raw minced meat beef stored at 4°C. Values with a different letter (a–d) of the same storage day are significantly different (*P* < 0.05); values with a different letter (A–E) of the same concentration are significantly different.

TBARS values resulted from their reaction with free amino acids, proteins, and peptides that are present in the minced meat, to form Schiff's bases, apart from the breakdown of the malonaldehyde due to tertiary degradation [37]. The increase of TBARS depended on the time of storage (Figure 5). Throughout the whole period of storage, TBARS value of control samples was greater than that of PHE groups. Remarkably, after 14 days, TBARS value of treated samples (BHT and PHEs) seemed to be significantly lower (*P* < 0.05) than in control and they were lower than 2 mg/kg (the acceptable sensory threshold limit) [38]. PV, CD, and TBARS were significantly (*P* < 0.05) lower in meat treated with PHE compared to control and BHT samples. The observed differences could be explicated by the presence of antioxidants in the PHE, which delay the lipid oxidation processes [11].

### 3.3. Sensory Analysis

Sensory results of minced beef meat were assessed during all the storage periods (Table 2). If the sensory score >5, the meat samples are considered suitable for human consumption [39]. Appearance, color, odor, and overall acceptability were given unacceptable scores by the 7^th^ day for control group, 10^th^ day for BHT group, and up to 14^th^ day for PHE groups. Oxidative changes, related to protein and lipid oxidation, and microbial growth influence the sensory quality, which can be enhanced by PHE addition. Earlier researchers have reported a similar trend of quality change [18, 33].

### 3.4. Chemometric Analysis

#### 3.4.1. PCA

**Figure 6:**
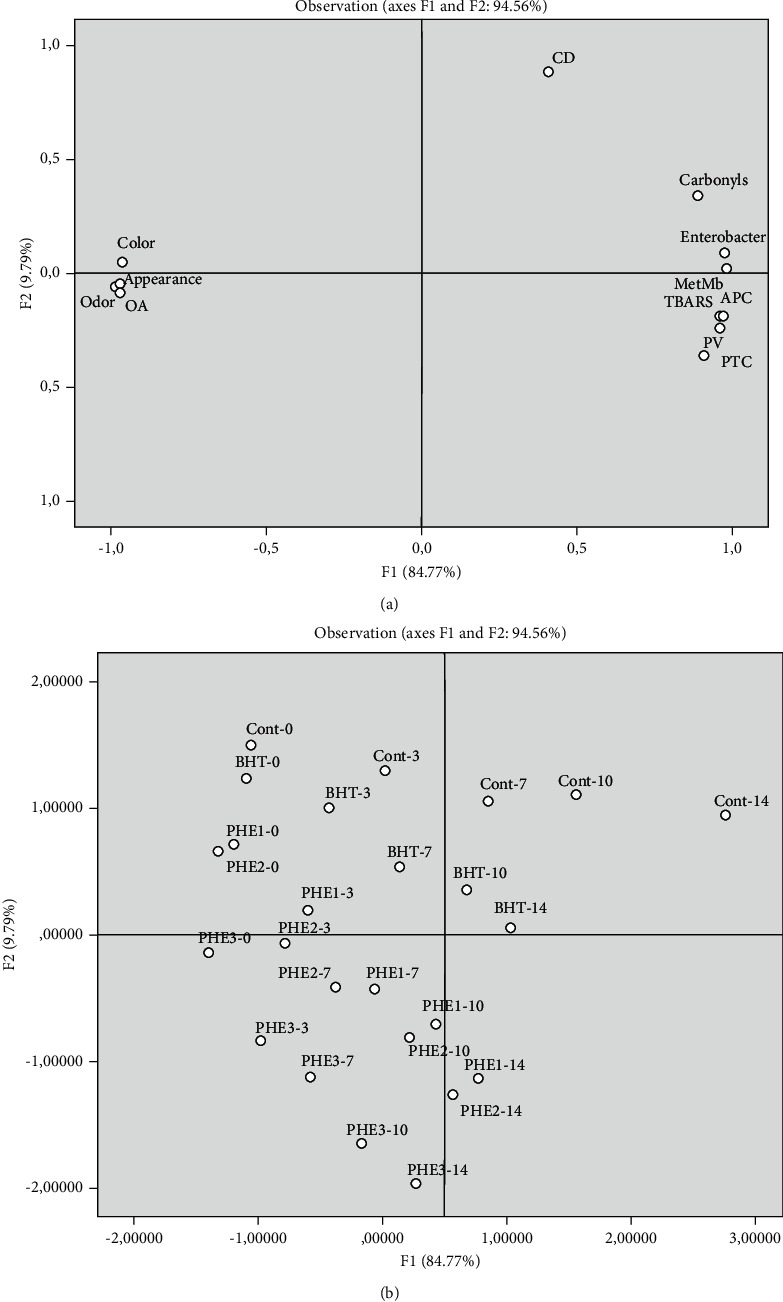
Principal component analysis (PCA) plots of physicochemical parameters, microbial loads, and sensory characteristics of different treated and untreated samples at each storage time: (a) variable-loading plot of PCA; (b) observation score plot of PCA.

In order to classify the studied samples according to the traits described above, PCA was used to confirm the cluster analysis results (Figure 6). Thus, we performed a PCA as a means to reduce the multidimensional structure of the data and to provide a two-dimensional map explaining the observed variance. The PCA accounted for 94.56% of the variance of the original data (Dim 1 : 84.77%, Dim 2 : 9.79%) (Figure 6(a)). A high correlation was observed between protein oxidation (carbonyls and MetMb), lipid oxidation (TBARS and PV), and microbial load (PTC, APC, and Enterobacteriaceae counts) which support the suggested interaction between lipid/protein oxidation and microbial growth. In addition, the increase of the storage time led to the disposition of the samples towards the right side of the PCA, which were designated by a high concentration of primary and secondary lipid and protein oxidation products and high microbial load (Figure 6(b)). In this regard, a recent research paper indicates that protein/lipid oxidation and microbial growth occur simultaneously [8]. Protein oxidation generates protein aggregates through the formation of disulfide bonds, which can delay with muscle proteolysis. This latter phenomenon induces the formation of small molecular components, principally composed by polypeptides, peptides, free amino acids, and amines and further enzymatic and chemical reactions leading to the release of nonprotein nitrogen compounds [40]. Furthermore, these authors demonstrated that aldehyde moieties from lipid oxidation products such as malondialdehyde can covalently bind to amino acid residues, resulting in indirect protein oxidation. On the other hand, the release of free fatty acids from meat lipids is facilitated by the synergistic action of endogenous enzymes and bacterial lipolytic enzymes [41]. With a shorter storage time (0–3 days), a significant and positive correlation was detected between control, BHT, PHE1, and PHE2 samples, and color. Meanwhile, remarkably, high scores of appearance, odor, and overall acceptability were closer with PHE3 at any storage time (0, 3, 7, 10, and 14 days) (Figure 6(b)). Interestingly, the use of PHE3 in minced beef meat prevents lipid/protein oxidation and allows a larger extent of proteolysis, leading to maintaining oxidation products and sensory attributes till the end of refrigerated storage time.

#### 3.4.2. Heat Map

**Figure 7:**
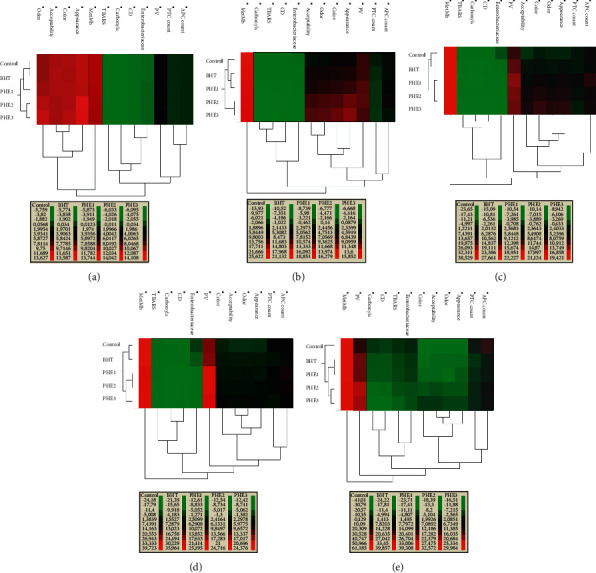
Agglomerative hierarchical cluster analysis (HCA) and heat map of physicochemical parameters, microbial loads, and sensory characteristics of different treated and untreated samples at each storage time periods: (a) 0 days, (b) 3 days, (c) 7 days, (d) 10 days, and (e) 14 days.

To summarize quantitative data of the samples regarding the lipid/protein oxidation, microbial growth, and sensory parameters at each storage time, we used the heat map represented in Figure 7. In this regard, the high number of extra correlations is corroborated by the heat map depiction of the correlation analysis. Each parameter was associated with a color: from green for low concentrations to red for high concentrations. At day 0, the present study indicated that MetMb % was the main contributor to the sensory attributes, which can also be influenced by the variation of lipid oxidation, carbonyl contents, and microbial growth. According to the color scale, at day 0, Figure 7(a) expresses four different clusters with a high similarity between control and BHT samples; moreover, PHE1, PHE2, and PHE3 presented dissimilarity in their composition. At days 3 and 7, dendrograms indicated the presence of four clusters: clusters I (Control), II (BHT), III (PHE1), and IV (PHE2 and PHE3). In these sampling days, it is clear that the nodes accumulation of CD, TBARS, and carbonyl contents was influenced by the growth of Enterobacteriaceae count (Figures 7(b) and 7(c)). In addition, dependency relation (PV-sensory attributes) was shown at day 3; however, at day 7, the relation was more significant between ((APC and PTC)-sensory attributes). At the end of storage, four groups were discriminated: clusters I (Control), II (BHT-PHE1), III (PHE2), and IV (PHE3). It should be noted that sensory traits were controlled directly by (APC and PTC), which also can indirectly be influenced by CD and carbonyl contents (Figure 7(e)). In this vein, Elhadef et al. [8], Fourati et al. [7], Nishad et al. [42], and Bouaziz et al. [43] studied the applicability of chemometrics for quality control and authentication of several types of meat and derived products (minced beef and turkey meat) incorporated by various plant (pomegranate peel, *Ephedra alata,* nutmeg, and citrus peel and date palm seeds) extracts.

## 4. Conclusion

The results of our study revealed that the addition of PHE can decrease lipid and protein oxidation and it can also increase microbiological stability and enhance sensory traits of raw minced beef meat stored at 4°C. During the storage time, a multitude of interactions among compounds derived from lipid/protein oxidation and microbial change contributed, therefore, to the intensification of sensory attributes. The results enabled discrimination of the meat samples, showing a great impact of the extract at three concentrations of PHE on the quality of meat samples. By the end of storage, factors including PV, APC, and PTC play a key role in modulating the sensory profile of the final product. Thus, industrial wastes like pistachio hull could be effectively used to extend the shelf life of refrigerated meat and derived products.

## Figures and Tables

**Table 1:** Effect of PHE on the microbial load of aerobic plate count (APC), psychrotrophic count (PTC), and Enterobacteriaceae count of raw minced meat beef stored at 4°C.

	Day	Control	BHT	PHE1	PHE2	PHE3
APC	0	2.22 ± 0.10^aA^	2.21 ± 0.09^aA^	2.23 ± 0.07^aA^	2.22 ± 0.10^aA^	2.22 ± 0.09^aA^
	3	5.34 ± 0.25^dB^	4.95 ± 0.23^bB^	5.17 ± 0.18^cB^	4.9 ± 0.22^bB^	4.07 ± 0.2^aB^
	7	7.65 ± 0.37^dC^	6.27 ± 0.31^bC^	6.86 ± 0.33^cC^	5.69 ± 0.27^aC^	5.5 ± 0.19^aC^
	10	8.24 ± 0.41^eD^	6.65 ± 0.30^bC^	7.07 ± 0.32^dCD^	6.47 ± 0.31^cD^	6.12 ± 0.27^aD^
	14	11.95 ± 0.59^dE^	7.19 ± 0.29^bcD^	7.27 ± 0.26^cD^	7.09 ± 0.34^bE^	6.68 ± 0.32^aE^

PTC	0	2.08 ± 0.08^aA^	2.05 ± 0.09^aA^	2.05 ± 0.07^aA^	2.02 ± 0.03^aA^	2.02 ± 0.04^aA^
	3	4.32 ± 0.17^dB^	3.72 ± 0.17^cB^	3.47 ± 0.17^bB^	3.39 ± 0.16^abB^	3.25 ± 0.10^aB^
	7	6.32 ± 0.31^eC^	5.54 ± 0.26^cC^	5.89 ± 0.22^dC^	5.03 ± 0.24^bC^	4.36 ± 0.16^aC^
	10	7.36 ± 0.29^dD^	5.78 ± 0.27^Bc^	6.1 ± 0.29^cC^	5.93 ± 0.28^bcD^	5.2 ± 0.22^aD^
	14	9.25 ± 0.42^dE^	6.18 ± 0.30^bD^	6.51 ± 0.31^cD^	6.26 ± 0.26^bE^	6.04 ± 0.25^aE^

Enterobacteriaceae counts	0	<1	<1	<1	<1	<1
	3	2.22 ± 0.1^dA^	1.49 ± 0.07^cA^	1.22 ± 0.06^bA^	1.15 ± 0.05^abA^	1.09 ± 0.04^aA^
	7	2.89 ± 0.14^cB^	1.92 ± 0.09^bB^	1.89 ± 0.09^bB^	1.33 ± 0.06^aA^	1.29 ± 0.06^aB^
	10	3.21 ± 0.16^dC^	2.29 ± 0.1^cC^	2.11 ± 0.09^bcB^	1.89 ± 0.1^bB^	1.51 ± 0.08^aC^
	14	3.54 ± 0.17^bD^	2.57 ± 0.12^bD^	2.41 ± 0.11^bC^	1.91 ± 0.1^aB^	1.88 ± 0.09^aD^

Values with a different letter (a–c) of the same storage day are significantly different (*P* < 0.05); values with a different letter (A–D) of the same concentration are significantly different.

**Table 2:** Effect of PHE on appearance, color, odor, and overall acceptability of raw minced meat beef stored at 4°C.

	Day	Control	BHT	PHE1	PHE2	PHE3
Appearance	0	6.63 ± 0.32^aE^	6.6 ± 0.31^aD^	6.61 ± 0.3^aC^	7.05 ± 0.31^Be^	7.05 ± 0.3^bD^
	3	6.2 ± 0.30^aD^	6.28 ± 0.29^aC^	6.5 ± 0.26^bC^	6.75 ± 0.27^cD^	6.9 ± 0.31^dD^
	7	5 ± 0.24^aC^	5.7 ± 0.24^bB^	6.1 ± 0.28^cB^	6.15 ± 0.24^cC^	6.21 ± 0.22^cC^
	10	4.2 ± 0.21^aB^	5.2 ± 0.22^bA^	5.26 ± 0.19^bA^	5.6 ± 0.26^cB^	5.69 ± 0.2^cB^
	14	3.1 ± 0.15^aA^	5.13 ± 0.14^bA^	5.19 ± 0.12^bA^	5.2 ± 0.21^bA^	5.26 ± 0.18^bA^

Color	0	6.55 ± 0.29^aE^	6.49 ± 0.23^aE^	6.6 ± 0.27^aD^	6.72 ± 0.29^aD^	6.78 ± 0.3^aC^
	3	6.1 ± 0.27^aD^	6.09 ± 0.22^aD^	6.25 ± 0.28^bC^	6.33 ± 0.24^bC^	6.54 ± 0.29^cC^
	7	5.75 ± 0.27^aC^	5.75 ± 0.19^aC^	6 ± 0.27^bB^	6.2 ± 0.22^cC^	6.5 ± 0.29^dC^
	10	4.8 ± 0.21^aB^	5.25 ± 0.22^bB^	5.25 ± 0.23^bA^	5.4 ± 0.22^cB^	5.8 ± 0.26^dB^
	14	3.2 ± 0.14^aA^	4.5 ± 0.22^bA^	5.15 ± 0.23^cA^	5.16 ± 0.19^cA^	5.5 ± 0.19^dA^

Odor	0	6.2 ± 0.21^aE^	6.19 ± 0.21^aC^	6.42 ± 0.29^bE^	6.75 ± 0.3^cE^	6.91 ± 0.31^dE^
	3	5.3 ± 0.2^aD^	5.9 ± 0.26^bC^	6.1 ± 0.27b^cD^	6.27 ± 0.28^cD^	6.3 ± 0.28^cD^
	7	5 ± 0.11^aC^	5.45 ± 0.21^bB^	5.75 ± 0.25^cC^	5.87 ± 0.24^cC^	6.1 ± 0.27^dC^
	10	4.15 ± 0.18^aB^	5.1 ± 0.22^bA^	5.4 ± 0.22^cB^	5.4 ± 0.2^cB^	5.6 ± 0.25 dB
	14	3.5 ± 0.15^aA^	5 ± 0.21^bA^	5 ± 0.22^bA^	5.16 ± 0.23^bcA^	5.33 ± 0.27^cA^

Overall acceptability	0	6.52 ± 0.29^aD^	6.52 ± 0.29^aD^	6.57 ± 0.22^aD^	6.6 ± 0.29^aD^	6.61 ± 0.28^aD^
	3	5 ± 0.2^aC^	5.95 ± 0.27^bcC^	5.8 ± 0.26^bC^	6.1 ± 0.27^cC^	6.18 ± 0.27^cC^
	7	4.3 ± 0.19^aBC^	5.63 ± 0.25^bB^	5.58 ± 0.24^bB^	5.75 ± 0.25^bB^	5.81 ± 0.26^bB^
	10	4 ± 0.18^Ab^	5.15 ± 0.1^bA^	5.25 ± 0.11^bcA^	5.33 ± 0.23^cA^	5.52 ± 0.21^dA^
	14	3.3 ± 0.11^Aa^	5 ± 0.11^bA^	5.14 ± 0.14^bcA^	5.25 ± 0.24^cA^	5.52 ± 0.22^dA^

Values with a different letter (a–c) of the same storage day are significantly different (*P* < 0.05); values with a different letter (A–D) of the same concentration are significantly different.

## Data Availability

The data used to support the findings of this study are included within the article. Raw data are available from the corresponding author upon request.
